# Content validity of mobility measures in arthrogryposis multiplex congenita: engaging clinicians and people with lived experience

**DOI:** 10.3389/fresc.2025.1576267

**Published:** 2025-08-04

**Authors:** Ahlam Zidan, Laurie Snider, Jaclyn Sions, Kristen Donlevie, Alexa Cirillo, Verity Pacey, Noémi Dahan-Oliel

**Affiliations:** ^1^ School of Physical and Occupational Therapy, Faculty of Medicine and Health Sciences, McGill University, Montreal, QC, Canada; ^2^ Department of Clinical Research, Shriners Hospitals for Children, Montreal, QC, Canada; ^3^ Department of Physical Therapy, University of Delaware, Newark, DE, United States; ^4^ Department of Occupational Therapy, Boston University, Boston, MA, United States; ^5^ Department of Health Sciences, Macquarie University, Sydney, NSW, Australia

**Keywords:** expert panel, lived experience, arthrogryposis, mobility, international classification of functioning, disability and health, content validity, patient-reported outcomes

## Abstract

**Introduction:**

Lower-extremity impairment is prevalent in children with Arthrogryposis multiplex congenita (AMC), frequently leading to mobility limitations. Without AMC-specific assessment tools, clinicians and researchers often employ tools that have not been formally validated for the AMC population. This study aims to establish the content validity of commonly used mobility measures in children with AMC following the COnsensus-based Standards for health Measurement INstruments (COSMIN) and the International Classification of Functioning, Disability, and Health (ICF) framework.

**Methods:**

Items from the measures “Functional Mobility Scale (FMS), Gillette Functional Assessment Questionnaire (FAQ), Functional Independence Measure for Children (WeeFIM), and Patient-Reported Outcomes Measurement Information System (PROMIS)” were linked to the ICF categories using the refined linking rules of the ICF. Three raters conducted independent linking, and inter-rater reliability was calculated using the Kappa coefficient. An expert panel consisting of people with lived experience, clinicians and researchers reviewed the ICF codes identified by the raters and evaluated the comprehensibility, relevance, and comprehensiveness of the four measures using the COSMIN standards. The Content Validity Index (CVI) and modified Kappa (k*) were calculated.

**Results:**

Inter-rater agreement was substantial [*κ* = 0.79, (95% CI: 0.78–0.84)]. Most concepts (84.4%) were linked to the “Activities and Participation” domain, with a limited representation of “Environmental Factors” (8.9%) and “Body Functions” (6.7%). The CVI and k* values for most measures indicated excellent content validity (0.91 to 1), except for the PROMIS Mobility Young Adult (≤0.82). The expert panel found that measures exhibited high comprehensibility and relevance, but comprehensiveness was insufficient. Most studied mobility measures missed concepts such as pain, fatigue, mobility aids, and compensatory strategies.

**Conclusions:**

FMS, FAQ, WeeFIM, and PROMIS (Parent Proxy/Pediatric) demonstrated good content validity. However, none of these measures fully address the full spectrum of mobility experiences in children with AMC. Incorporating missing concepts, such as environmental challenges, compensatory strategies, and pain, into existing or newly developed assessment tools is essential for providing a more holistic evaluation of functional mobility. Doing so will support more comprehensive clinical assessment, improve outcome tracking, and enhance care for children living with AMC.

## 1 Introduction

Arthrogryposis multiplex congenita (AMC) encompasses a spectrum of non-progressive congenital conditions characterized by multiple contractures present at birth, leading to impaired muscle and joint function. AMC affects 1 in 3,000 live births, with fetal akinesia implicated in its pathogenesis, which disturbs joint formation (1, 2). Lower-extremity impairment is prevalent in 56%–90% of children with AMC, frequently leading to mobility difficulties and adversely restricting engagement in recreational and community activities (3–5).

The World Health Organization's International Classification of Functioning, Disability, and Health (ICF) defines mobility as “moving by changing body position or location or by transferring from one place to another, by carrying, moving or manipulating objects, by walking, running or climbing and by using various forms of transportation” (6). Effective mobility facilitates environmental exploration, peer interaction, and the acquisition of independence, all of which are critical for achieving developmental milestones (7). This necessitates clinicians providing individualized assessments to effectively track and support functional development. Without AMC-specific assessment tools, clinicians and researchers often employ tools initially developed for other populations, such as cerebral palsy (CP). Frequently cited mobility measures within the AMC community are the Functional Mobility Scale (FMS) (8–11), Gillette Functional Assessment Questionnaire (FAQ) (11, 12), Functional Independence Measure for Children (WeeFIM) (9–12), and the Patient-Reported Outcomes Measurement Information System (PROMIS) (8, 10, 11). FMS, FAQ, and PROMIS are patient-reported outcome (PRO) measures, while the WeeFIM is a clinician-reported outcome (ClinRO) measure. To date, none have been formally validated for the AMC population.

Measurement validity is contingent upon specific population and contextual implementation rather than being an inherent characteristic of the measure (13). The need for valid assessment tools that measure constructs relevant to AMC, such as pain and mobility, was highlighted at the 3rd International Symposium on Arthrogryposis in Philadelphia, 2018 (14). Ensuring content validity is a prerequisite before evaluating other measurement attributes (15). Content validity is defined by the relevance and representativeness of assessment elements to the intended construct within a specific context (16). Ensuring the relevance and applicability of mobility measures for children with AMC necessitates the inclusion of diverse perspectives, particularly those of individuals with lived and clinical experience. Incorporating the insights of individuals with AMC, families, clinicians, and researchers enhances the comprehensiveness of mobility assessments by identifying critical but often overlooked aspects of mobility, such as compensatory strategies, environmental barriers, and the psychosocial impact of mobility limitations (17, 18). This study integrates an expert panel of researchers, clinicians, individuals with AMC and caregivers to assess the comprehensibility, relevance, and comprehensiveness of commonly used mobility measures, ensuring that these tools accurately capture mobility challenges and priorities for individuals with AMC from multiple perspectives.

The primary aim of the current study was to evaluate the content validity of commonly used mobility measures for children with AMC by estimating the extent to which the mobility dimensions of these children are encapsulated within these measures in accordance with the ICF framework. We hypothesize that the mobility measures will have good to excellent content validity.

## 2 Methods

The content validation process of PRO or ClinRO measures should involve defining the construct under study based on theoretical and conceptual foundations and selecting an expert panel to evaluate the relevancy and comprehensiveness of the measure to the construct and context (19, 20). Following these recommendations, the content validity of four mobility measures was examined using the ICF linking rules and the COnsensus-based Standards for the selection of health Measurement INstruments (COSMIN) standards (21). The ICF linking rules offer a standardized guideline for linking health concepts to the ICF to describe health and functioning, ensuring consistency and using a common language, facilitating a comprehensive content assessment of the outcome measure (22).

The COSMIN methodology was developed in an international Delphi study to evaluate the content validity of patient-reported outcome (PRO) measures (21, 23). We applied the same content validity principles to ClinRO measures, with minor adaptations as needed. To ensure content validity in a measure based on the COSMIN standards, three criteria must be met: the items are relevant to the construct and population of interest (Relevance); comprehensively cover all aspects of the construct being measured (Comprehensiveness); and the items, responses, and instructions are clear and appropriately worded (Comprehensibility) (21, 24).

### 2.1 Instruments

The selection of the mobility measures was based on their widespread use in pediatric rehabilitation for children with mobility impairments, including neuromuscular and orthopedic conditions, as identified in recent literature reviews (11, 25, 26). No AMC-specific outcome measures currently exist, and each of the selected tools captures different aspects of mobility, such as walking distance, independence in transfers, or the use of assistive devices.

• FMS is a 6-level measure that classifies functional mobility depending on the assistance required for a child's mobility at three specific distances: 5, 50, and 500 metres (27). Its psychometric properties have been demonstrated in children with CP (28, 29).
• The walking scale of FAQ assesses a child's walking (ambulation) abilities in different environments and terrains on a 10-level ordinal scale (30). It has been proven valid for children with CP and reliable for the AMC population (31, 32).
• WeeFIM is a reliable measure that assesses independence and functional skills in children during basic daily living (33). Only the mobility domain, including transfers and locomotion, was assessed for this study. The transfers consist of three subscales: Chair/Wheelchair, Toilet, and Tub/Shower, while the locomotion consists of Walk/Wheelchair/Crawl and Stairs subscales. WeeFIM consists of a normative value set (34), and its items are rated through observation or interview on a 7-level scale. The WeeFIM mobility domain showed decreased scores in children with AMC compared to norms (9, 35).
• PROMIS, the short forms of the PROMIS physical function-mobility domain, were assessed in this study. PROMIS consists of three versions based on the interviewee type: the parent proxy and the pediatric versions (Bank v2.0-SF8a) were completed by the caregiver of children 5–17 years and by capable youth 8–17 years, respectively, and the Young Adult version (Bank v2.0-SF8b) by a young adult 18–21 years (36). It was validated in children with Talipes Equinovarus and CP (37, 38), and commonly used for children with AMC (39, 40). The scores of the PROMIS are on a T-score metric (mean 50, standard deviation 10), and higher T-scores represent better functioning (41).

### 2.2 Data collection and analysis

Development of Assessment Forms: Two content validity assessment forms were created using Microsoft Excel 365: the ICF-linking form and the comprehensiveness, relevance, and comprehensibility form. The former was used to link the items of the measures to the ICF. The latter form was created to comment on the ICF-linking process and assess content validity of the mobility measures based on the COSMIN guidelines (i.e., comprehensibility, relevance, and comprehensiveness).

**Figure 1:**
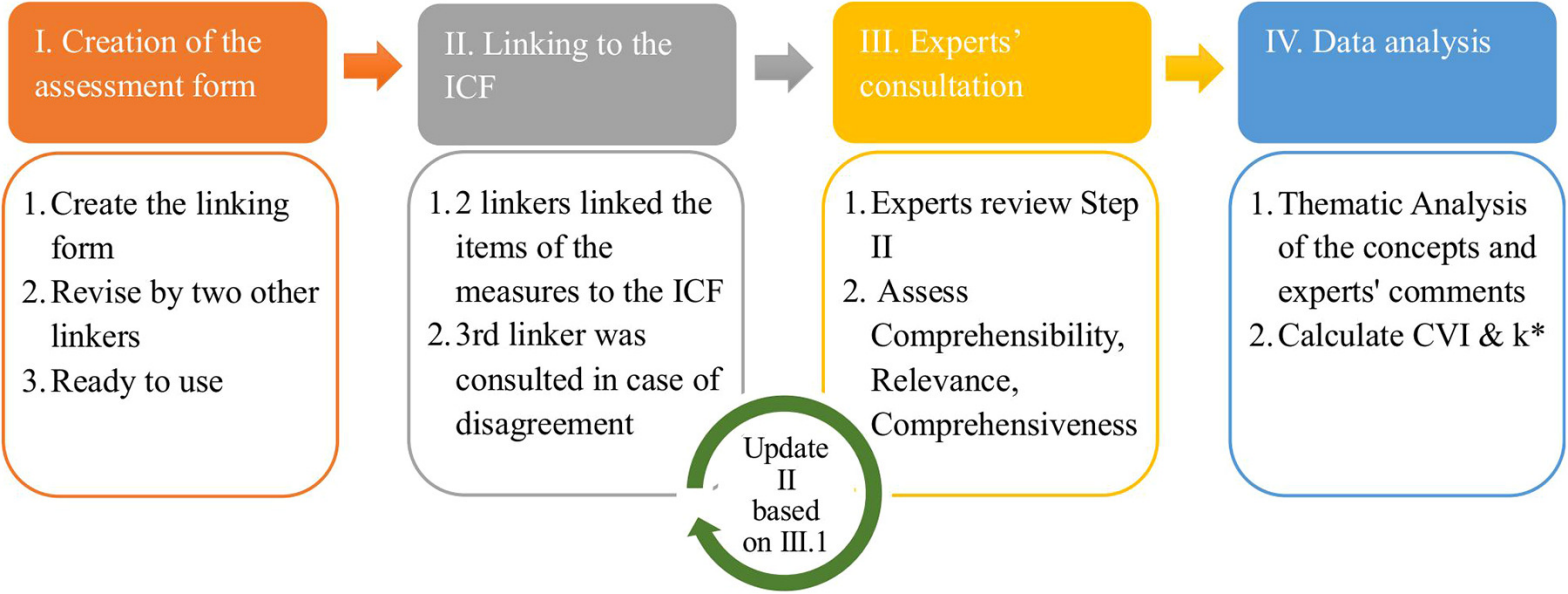
The content validation process of the four mobility measures.

ICF-based linking procedure: The linking process of the measures was performed by three raters (AZ, AC, and NDO) following the refined linking rules and steps proposed by Cieza et al. (22). Before starting the linking process, the raters participated in an E-learning tool recommended by the World Health Organization (https://www.icf-elearning.com) to acquire foundational knowledge of the conceptual and taxonomical fundamentals of the ICF. Additionally, the raters performed a mock linking session to align perspectives and ensure all raters had a comparable understanding of the linking rules. Using the ICF-linking form, two raters (AZ and AC) independently linked each item to the ICF. The third rater (NDO) determined whether an agreement had been reached and was consulted in case of disagreement. A visual summary of the content validation process is provided in Figure 1 to support clarity.

Meaningful concepts were identified from each item of the outcome measures based on steps 2 and 3 of the ICF linking rules (22). A concept was defined as one meaningful entity; one or more concepts could be identified from a single item. To identify meaningful concepts of the FMS and FAQ, we considered both the items and the responses to these items since the responses were richer in concepts than the items. The meaningful concepts were then linked to the most precise ICF category related to Body Functions, Body Structures, Activities and Participation, and Environmental Factors. Concepts were also linked to Personal Factors (pf) when needed. If a concept was too general or vague or was beyond the scope of the ICF, the categories “nd” (not definable) or “nc” (not covered) were assigned, respectively.

Inter-rater reliability: Inter-rater reliability was calculated using Kappa coefficient (*κ*) to measure the level of agreement between the two main raters (AZ and AC). The Kappa coefficient was calculated for 25% and 75% of the linked items using STATA software. A *κ* value of ≥0.61 suggests good agreement between raters (42).

Expert panel: Five researchers with academic/clinical expertise in AMC and measurement, and two with academic/clinical and lived experience with AMC, formed an expert panel to assess the comprehensibility, relevance, and comprehensiveness of the mobility measures using the second form. Experts were identified based on their clinical or research expertise in AMC, prior involvement in outcome measure development or evaluation, and/or lived experience with AMC. Recruitment was conducted through the research team's clinical and academic networks to ensure diverse and informed perspectives. Panel members included physiotherapists (*n* = 3) and occupational therapists (*n* = 4). Four experts had over 10 years of academic experience with Associate Professor rank.

Relevance and comprehensibility of items were rated on a 4-point scale, with 1 indicating that the item was irrelevant or did not match the responses and 4 indicating high relevance and a good match between the item and response options (43). Comprehensiveness was evaluated based on detailed feedback regarding whether any key concepts were missing from the measure. Through an open-ended question, experts were invited to suggest additional ICF categories or concepts judged relevant for the AMC population, explain vague or redundant items, and indicate agreement or dissent with the identified meaningful concepts by the two raters (AZ and AC).

Data analysis was conducted using Microsoft Excel to calculate the number of ICF categories identified for each measure. Each ICF category was counted only once per measure, even if the same category appeared in multiple items. The Content Validity Index (CVI) was calculated at both the item (I-CVI) and scale level (S-CVI). S-CVI is the average of I-CVIs of the measure. Higher CVI values indicate greater content validity. The S-CVI is influenced by the number of experts, with a greater number of experts increasing the likelihood of a lower S-CVI due to the requirement for universal agreement (UA), where an item is given a score of 1 if all experts agree, and 0 otherwise (44). For a measure to be deemed excellent regarding content validity, an I-CVI of 0.78 or higher and a S-CVI of 0.90 or higher are recommended alongside a solid conceptual framework, which was assessed through the linking to the ICF (43, 44).

To address CVI limitations, the *modified Kappa* (k*) was used to account for chance agreement, ensuring that the validity index (CVI) reflects true expert consensus beyond what might occur by chance. k* values were categorized as fair (0.40–0.59), good (0.60–0.74), or excellent (≥0.74). These calculations were performed following the guidelines proposed by Almanasreh et al. (43).

A thematic analysis of the meaningful concepts and the experts' comments regarding the relevance, comprehensibility, and comprehensiveness of the mobility measures was done using an inductive coding approach. Two researchers (AZ and AC) independently reviewed the open-ended responses, generated initial codes, and organized these codes into preliminary themes. These themes were iteratively refined through discussion and consensus with a third author (NDO) to enhance analytical rigor and ensure that emerging themes accurately reflected expert perspectives. This approach identified patterns and themes to explain agreement and/or disagreement patterns and gain insight into the missing constructs deemed relevant to the AMC population. Ethical approval was not required because this study did not involve human subjects as participants.

## 3 Results

### 3.1 Inter-rater reliability

The agreement between raters was substantial, with a *κ* of 0.79 (95% CI: 0.78–0.84), indicating that the linking process yielded consistent results (42).

### 3.2 Linking to the ICF

**Figure 2:**
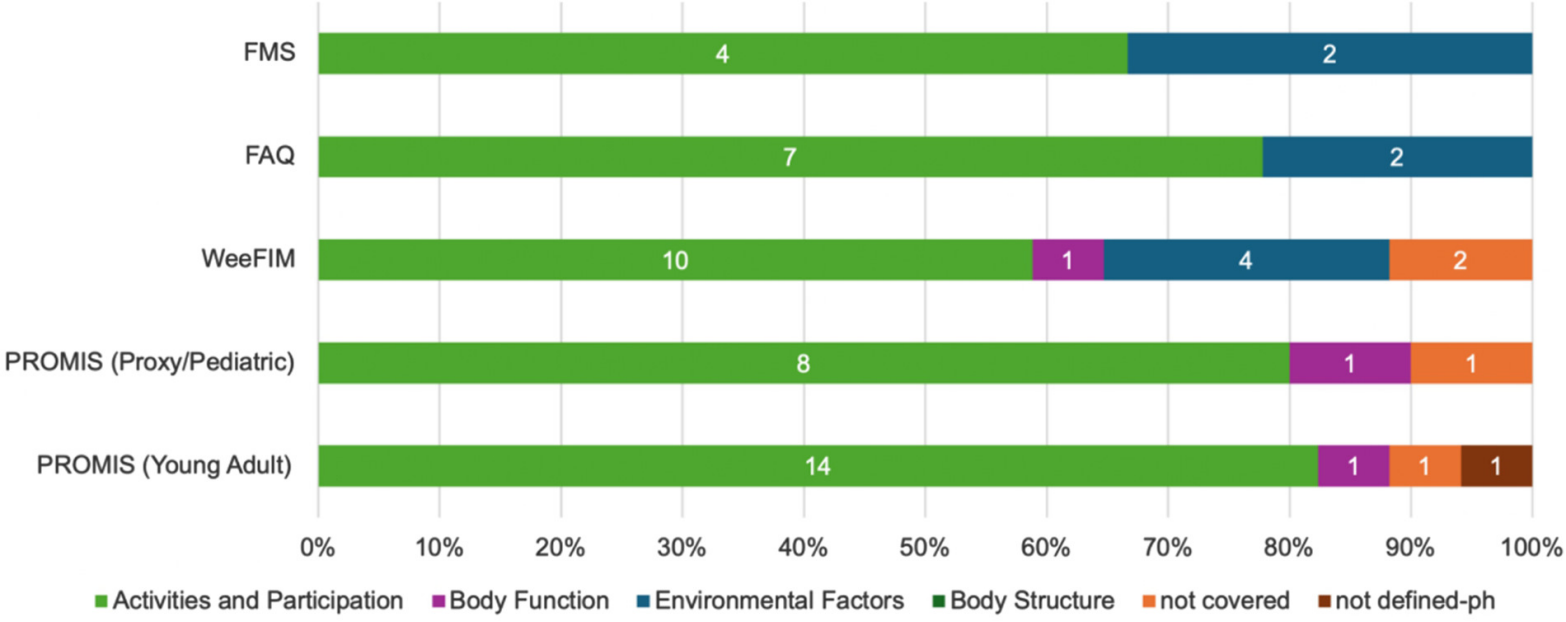
Distribution of ICF categories (# on the bars) and domains across mobility measures.

**Figure 3:**
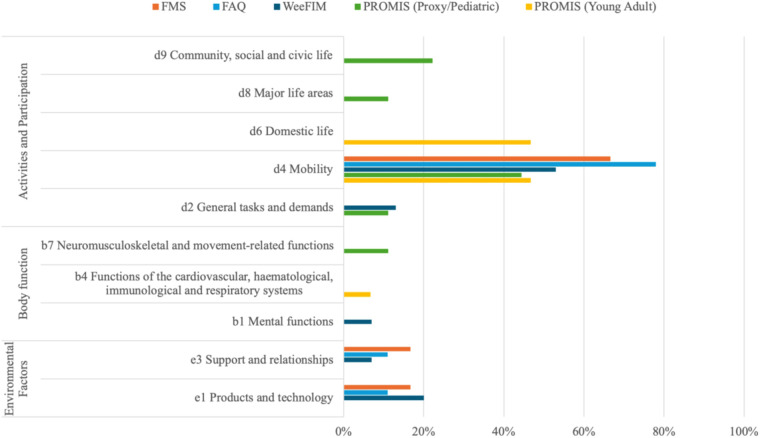
Distribution of ICF domain and chapters across mobility measures (the percentages represent the proportion of total items in each measure linked to each ICF domain).

A total of 50 meaningful concepts emerged from the 69 items identified across the four mobility measures. Of these, 45 meaningful concepts were successfully linked to ICF categories. Four concepts were not covered by the ICF, and one concept was assigned “not defined-physical health” (nd-ph). A full listing of main concepts, items, linked ICF categories, and themes is presented in Table 1. These concepts were distributed across three ICF domains: Activities and Participation, Body Functions, and Environmental Factors. A detailed analysis of domain representation revealed that 84.4% of the concepts were linked to Activities and Participation, 6.7% to Body Functions, and 8.9% to Environmental Factors (Figures 2, 3). These meaningful concepts were grouped under 13 themes (Supplementary Table S1).

**Table 1:** ICF categories, meaningful concepts, themes per mobility measure.

Measures	Themes	Meaningful concepts [item/subscale #]	ICF categories
FMS	Moving Around	Moving around short distances [5 m main question, response 6]	d4600
		Moving around in and between classes at school [50 m main question, response 6] Moving around long distances [500 m main question, response 6]	d4601
	Assistance and Independence	Walking with assistance [response 5]	d465 e399
		Need of assistive device [responses 1–4]	e1201
	Crawling	Crawling [response C]	d4550
FAQ	Walking	Walking [main question, responses 1–10]	d450 d4500 d4501 d4502
		Walking with assistance [main question, responses 2, 7, 8, 9]	d465 e399
	Stair Navigation	Climbing [responses 9, 10]	d4551
	Assistance and Independence	Need of assistive device [main question, responses 6]	e1201
	Basic Movements and Postures	Running [responses 9, 10]	d4552
WeeFIM Mobility Subscales: 1. Chair wheelchair 2. Toilet 3. Tub, Shower 4. Walk 5. Wheelchair 6. Crawl 7. Stairs	Walking	Walking [4] Perform less than 25% of the effort required for walking [4] Perform 75% or more of the effort to walk 150 feet [4]	d4500 d4501
	Assistance and Independence	Need of assistive device [1, 2, 3, 4, 7]	e1201 e1151 e1503
		Need help [1, 2, 3, 4, 5, 7]	e399
		No need of assistance [4, 5, 7]	d2102
		Assistance from one helper [1, 2, 3, 4, 5, 7] Assistance from two helpers [1, 2, 3, 4, 5, 7]	d2108
		Transfer/Travel with assistive device [1, 2, 3, 4, 5, 7]	d465
	Wheelchair Mobility	Perform 25% of the effort to travel with a wheelchair for 150 feet [5] Perform 75% of the effort to travel with a wheelchair for 150 feet [5]	d4208
	Basic Movements and Postures	Sitting [1, 2]	d4103
	Transfer Tasks	Transfer [1, 2] Perform 25% or more of transfer tasks [1, 2] Perform 50% or more of transfer tasks [1, 2] Perform 75% or more of transfer tasks [1, 2]	d4200
	Stair Navigation	go up and down (12–14) stairs [7] go up and down (4) stairs [7] Go up and down fewer than 4 stairs [7] Perform 75% or more of the effort to go up and down 12–14 stairs [7]	d4551
	Time and Effort	Time to go up and down [7] Time to walk/transfer [1, 2, 3, 4]	b1470
	Crawling	Crawling [6]	d4550
	Miscellaneous Mobility Aspects	Safety concern [1, 2, 3, 4, 5, 7]	nc
		Distance [6]	nc
PROMIS (Proxy/Pediatric) Mobility	Physical Activities and Recreation	Sports and exercise [1]	d9201
		Keep up playing with other kids [3]	d469 d8803
		Do the activities they enjoy [8]	d9209
	Miscellaneous Mobility Aspects	Moving legs [4]	b7600
	Assistance and Independence	Standing without help [5] No need of assistance [7]	d2102
	Basic Movements and Postures	Standing on tip toes [6]	d4108
		Getting up from the floor [2]	d4104
	Stair Navigation	Walk upstairs without holding on to anything [7]	d4551
	Generic “Physical Labor and Health”	Physical ability [8]	nc
PROMIS (Young adult) Mobility	Household Activities and Personal Errands	Doing chores [1]	d640
		Run errands and shopping [4]	d620 d6200 d6201
		Doing moderate housework [6]	d640 d4300 d4301 d4302 d6402
		Doing heavy work around the house [8]	d430 d6102 d6402
		Lifting or carrying groceries [7]	d449 d4300 d4301 d4302
	Time and Effort	Normal pace [2]	nc
	Walking	Going for a walk of at least 15 min [3]	d4501
	Generic “Physical Labor and Health”	Doing two hours of physical labor [5]	b4550
		Physical health [5, 6, 7, 8]	nd-ph

ICF, international classification of functioning, disability and health; FMS, functional mobility scale; FAQ, gillette functional assessment questionnaire; WeeFIM, functional independence measure for children; PROMIS, patient-reported outcomes measurement information system; nc, not covered; nd-ph, not defined-physical health.

The identified concepts were then linked to 40 ICF categories, of which 33 categories represented Activities and Participation (mainly the mobility chapter), three Body functions, and four Environmental factors.

• FMS: Six meaningful concepts were identified and linked to six unique ICF categories. The meaningful concepts were grouped under three themes.
• FAQ: Five meaningful concepts were linked to nine ICF categories. These concepts were categorized into four themes.
• WeeFIM mobility: Twenty-five concepts were identified, grouped under nine themes, and linked to 15 ICF categories. Two concepts, “Safety” and “Distance”, emerged from “Is there a concern for safety when the child transfers/ walks/ goes up and down stairs?”, “Does the child crawl at least 50/30/15 feet (15 m)?” items, respectively, were not covered by the ICF and assigned “nc”.
• PROMIS mobility: Ten meaningful concepts were identified from the Parent Proxy and Pediatric versions of PROMIS, grouped into six themes and linked to nine ICF categories. In both versions, the “Physical Ability” concept, which emerged from the item “My child has been physically able to do the activities he/she enjoys most”, was not covered by the ICF and assigned “nc”. In the Young Adult version, ten concepts were identified, with 15 ICF categories linked. One concept, “normal pace”, which emerged from the item “Are you able to go up and down stairs at a normal pace?”, was not covered by the ICF and assigned “nc”, and the concept “physical health”, emerged from last 4 items, was very generic and assigned not defined “nd-ph”. The concepts were grouped into five themes. See Table 1 for a detailed description.

### 3.3 Comprehensibility, relevance, and comprehensiveness

COSMIN guidelines recommend that the expert panel should include experts with “knowledge of the construct of interest; experience with the target population; and knowledge or experience with PROM development and evaluation” (21). Our expert panel met all these criteria.

• Comprehensibility: When asked, “Do the response options match the question (item)?”, expert ratings ranged between 3 and 4 out of 4 for all measures, indicating that the questions and responses were generally well-aligned (UA = 1).
• Relevance: The I-CVI of all items among the four measures ranged from 0.71 to 1, of which the last four items of the PROMIS Young Adult scored the lowest. Most items of PROMIS Young Adult and WeeFIM scored ≤0.86, influencing their overall CVI. The S-CVI for all measures ranged from 0.91 to 1, reflecting excellent content validity based on the relevancy to both the mobility construct and the AMC population, except the PROMIS/Young Adult, which scored 0.82. Similarly, k* values indicated excellent content validity for all items (≥0.85), except for the last four items of the PROMIS Young Adult measure (0.66) (Table 2). Supplementary Table S2 presents a detailed CVI.
• Comprehensiveness: Experts identified several missing concepts that should be considered to fully capture mobility in children with AMC (see Table 3). These included pain, fatigue, type and use of orthoses (e.g., AFOs/KAFOs), wheelchair type (manual or powered), level and type of assistance required, outdoor mobility, mobility on different terrains, floor mobility strategies such as scooting or rolling, environmental factors (e.g., chair height, curbs), and compensatory strategies.
• Through the open-ended questions, the experts identified content validity issues among the four mobility measures. For example, irrelevant items or concepts to the mobility construct for children with AMC, lack of clarity, redundancy, or less clinically applicable concepts (Table 3).

**Table 2:** Content validity index of the mobility measures.

Measure	Relevance to the construct		Relevance to the population	
	S-CVI	I-CVI range	S-CVI	I-CVI range
FAQ	1	1	1	1
FMS	1	1	1	1
WeeFIM (Mobility)	0.91	0.86–1	0.89^a^	0.86–1
PROMIS (Mobility)				
Proxy/Paediatric	0.93	0.86–1	0.95	0.86–1
Young Adult	0.82^a^	0.71^a^–1	0.89^a^	0.86–1

^a^ Values less than the recommended.

I-CVI, item content validity index; S-CVI, scale content validity index; FMS, functional mobility scale; FAQ, gillette functional assessment questionnaire; WeeFIM, functional independence measure for children; PROMIS, patient-reported outcomes measurement information system.

**Table 3:** Expert opinion on the content coverage for each mobility measure.

Measure	Content issue and *Items*	Description
FMS	Irrelevant to the population. *Crawling: Child crawls for mobility at home (5* *m).*	In the FMS-5 m scale, the only floor mobility was considered is crawling, which might not be relevant to many of AMC patients who are unable to crawl and compensate with scooting or rolling.
	Missing concepts	1. Floor mobility (scooting, rolling, and getting off the floor) 2. Use of orthoses such as AFOs and KAFOs 3. Wheelchair type (manual or powered) 4. Pain level during a task 5. Fatigue level during a task 6. Time required to complete mobility tasks 7. Safety
	Additional comments *How does your child move around for short distances in the house?*	The FMS question is general to some extent and can cover different aspects of mobility; however, the responses cover only one aspect of mobility: the level of assistance needed to move around.
FAQ	Missing concepts	1. Floor mobility (crawling, scooting, rolling, and getting off the floor) 2. Use of braces such as AFOs and KAFOs 3. Wheelchair type (manual or powered) 4. Level and type of assistance 5. Pain level during a task 6. Fatigue level during a task 7. Time required to complete mobility tasks
	Additional comments *Please choose the answer below that best describes your child's usual or typical walking abilities (with the use of assistive devices typically used).*	The FAQ question relates more to the child's walking abilities; however, the responses cover climbing and running abilities
WeeFIM	Irrelevant to the population	Crawl and Stairs subscales: for AMC population, scooting is very relevant and should be considered here; some children with AMC could scoot if unable to crawl or climb stairs.
	Missing concepts	1. Floor mobility (scooting, rolling, and getting off the floor) 2. Wheelchair type (manual or powered) 3. Pain level during a task 4. Fatigue level during a task 5. Extent of upper limb involvement in mobility tasks 6. Environmental factors (e.g., chair type and height) 7. Compensations (e.g., climbing stairs by scooting)
	Less applicable clinically *Transfer: #6 Chair/Wheelchair, #5 Toilet (Does the child require assistance from two helpers to transfer?)*	Some items mentioned access to two helpers, which is not clinically available usually and replaced with technology, the question should mention “or a helper with an assistive device support”
	Additional comments *Locomotion: #1 Wheelchair [Does the child need help to travel 150 feet (45* *meters) in a wheelchair?]*	Item 1, under Locomotion: Wheelchair, should consider the environment, which could give more detailed mobility status to guide the treatment plan, for example: need help only when the environment has curbs, or crowded shopping mall.
PROMIS Proxy/Pediatric	Irrelevant to the construct *#3 My child/I could keep up when he/she/I played with other kids* *#4 My child/I could move his/her/my legs* *#6 My child/I could stand up on his/her/my tiptoes* *#8 My child/I has/have been physically able to do the activities he/she/I enjoy most*	Item 3: It is more about the child's engagement than mobility, and engagement could be influenced by other factors such as (pain, fatigue, psychological, and social skills) not related directly to mobility, so this item is more representative of the child's social behaviour than mobility. Item 4: more relevant to the movement construct than mobility, and it should be put in a context of moving their legs to accomplish a task like walking. Item 6: standing on tip-toes is not important for mobility especially for children with disabilities, it is more relevant to a higher level of balance and advanced activities like in professional sports. Item 8: is not clear if the physical ability assessed here is relevant to activities that must involve lower limb mobility, because activities that children might enjoy like drawing will require physical ability relevant to upper limb, which is not a relevant indicator of mobility construct.
	Irrelevant to the population *#6 My child/I could stand up on his/her/my tiptoes*	Item 6: irrelevant since clubfoot deformity and the use of orthotics are common in children with AMC.
	Lack of clarity *#3 My child/I could keep up when he/she/I played with other kids*	Item 3: unclear which type of playing is assessed here. Some activities involve only the upper limbs.
	Redundancy *#1 My child/I could do sports and exercise that other kids his/her/my age could do*	Items 1 and 8: redundant (pleasure and recreation)
	Missing concepts	1. Floor mobility (crawling, scooting, rolling, and getting off the floor) 2. Outdoor mobility 3. Mobility needs in daily life functioning (e.g., toileting, dressing) 4. Extent of upper limb involvement in mobility tasks 5. Safety
	Additional comments *#8 My child/I has/have been physically able to do the activities he/she/I enjoy most*	Item 8: a huge percentage of the AMC population will be able to do activities they enjoy with adaptations and assistive devices, however, neither the question nor the responses consider that.
PROMIS Young Adult	Irrelevant to the construct *#1 Are you able to do chores such as vacuuming or yard work?*	Item 1: doing chores relates to abilities beyond mobility e.g., cognition and upper limb coordination and fine motor skills. The question could assess the impact of upper limb function on individual ability to do chores more than their ability to move around and being mobile. For example, vacuuming and planting can be done even when you cannot walk and just during sitting.
	Lack of clarity *#2 Are you able to go up and down stairs at a normal pace?* *#4 Are you able to run errands and shop?* *#5 Does your health now limit you in doing two hours of physical labor?* *#6 Does your health now limit you in doing moderate work around the house like vacuuming, sweeping floors or carrying in groceries?* *#7 Does your health now limit you in lifting or carrying groceries?* *#8 Does your health now limit you in doing heavy work around the house like scrubbing floors, or lifting or moving heavy furniture?*	Item 2: it is not clear what a “normal pace” refers to? Is it compared to same-age child without AMC? Item 4: is not a good reflector of an individual's mobility, if the patient has cognition or upper limb impairments. Also, because shopping can be done nowadays online while sitting or lying down, it is important to specify the question to reflect the involvement of lower limb and moving around to accomplish this task (running errands and shopping). Item 5: needs to clarify what physical labor and health (physical or also mental) exactly refer to here, to ensure the interviewee is understanding and answering correctly what reflects the child's mobility. Items 6, 7, 8: needs to clarify what health (physical or also mental) exactly refers to here and to distinguish between if the limitation is because of lower limb impairment or other reasons (pain level, respiratory function, fatigue, hand and upper limb function, and psychological issues)
	Redundancy	Items 6 and 7: redundant (carrying groceries)
	Missing concepts	1. Level and type of assistance 2. Outdoor mobility 3. Walking on different terrain 4. Use of orthoses such as AFOs and KAFOs 5. Pain level during a task 6. Fatigue level during a task 7. Safety

AMC, arthrogryposis multiplex congenita; FMS, functional mobility scale; FAQ, gillette functional assessment questionnaire; WeeFIM, functional independence measure for children; PROMIS, patient-reported outcomes measurement information system; AFOs, ankle-foot orthoses; KAFOs, knee-ankle-foot orthoses.

## 4 Discussion

The purpose of this study was to assess how well the content of commonly used outcome measures captures the mobility construct for children living with AMC. To our knowledge, this is the first study to evaluate the content validity of the FMS, FAQ, PROMIS, and WeeFIM specifically for the AMC population.

Engaging individuals with lived experience and clinicians provided critical insights into the content validity of mobility measures. This inclusive approach ensured that assessments were not based solely on theoretical frameworks but also reflected real-world experiences and functional challenges. It aligns with broader patient-oriented research efforts that prioritize meaningful input from those directly affected by a condition, thereby improving the applicability and acceptability of assessment tools (45).

Comprehensive outcome measures must consider mobility as a multidimensional construct influenced by physical, environmental, and psychosocial factors (46, 47). Our findings indicate that most items were linked to the ICF Activities and Participation domain. Although the Environmental Factors domain was represented in three measures (FMS, FAQ, WeeFIM), many important concepts, such as powered mobility and terrain challenges, were absent. Only WeeFIM and FAQ addressed safety in mobility tasks, yet no ICF category could be linked to this concept. Despite their widespread use, the four measures do not fully capture the unique mobility challenges faced by children with AMC. Key influences on mobility in this population, including orthotic device use, types of mobility aids, compensatory strategies (e.g., scooting instead of crawling), pain, and fatigue, were either underrepresented or omitted. These factors map to ICF categories such as e1151 (Assistive products and technology for personal use in daily living), b280 (pain), and b4552 (Fatiguability), providing clinicians with a framework to explore these areas during assessment.

Our expert panel emphasized the importance of incorporating pain and the type of mobility aid into mobility assessments for children with AMC. Pain has been shown to significantly affect mobility, participation, and engagement, and is one of the most common reasons for orthopedic referrals in this population (10, 48, 49). Individuals with AMC often prefer powered mobility aids (wheelchairs/scooters), especially outdoors (50, 51). Using a wheelchair was associated with lower health-related quality of life for children with AMC (51).

Of the tools evaluated, the WeeFIM is the only measure with a specific subscale for assessing wheelchair mobility. Yet, its overall content validity index (CVI) was below 0.90. Experts noted that its items do not adequately address the type of wheelchair (manual vs. powered) or environmental terrain, factors that are especially relevant for outdoor mobility (52).

Given these limitations, clinicians may consider supplementing existing tools with additional measures or combining complementary instruments to better capture the multidimensional nature of mobility in AMC. For example, pairing the FMS, which evaluates walking ability, assistive device use, and distance, with the WeeFIM mobility domain, which captures transfers and wheelchair use, may provide a more comprehensive view of function. Clinicians may also explore other relevant domains from PROMIS or WeeFIM, such as upper body function, daily activities, or pain interference, depending on the child's specific presentation and needs (53). For those specifically interested in evaluating lower-extremity pain, the Oswestry Disability Index may provide useful insights, although it has been validated only in adults with AMC (54).

In parallel, our team is currently leading the development of a Pediatric Core Outcome Set for AMC through an international multi-stakeholder consensus process, registered with the COMET initiative (55). This outcome set will help guide clinicians in selecting additional tools relevant to the AMC population, fostering more standardized and inclusive care practices. Additionally, broader functional assessments, such as the Pediatric Evaluation of Disability Inventory—Computer Adaptive Test (PEDI-CAT), can be used to evaluate mobility within the context of everyday activities and participation (4).

The selection of a mobility measure should also be informed by the child's functional level. Non-ambulatory children with AMC will require a measure that assesses their transfers, standing, sitting, and wheelchair skills, which could reflect their functional status in daily life activities (56–58). Based on our findings, WeeFIM and PROMIS (Proxy/Pediatric) covered concepts such as standing, sitting, transfers, and wheelchair mobility. Based on our findings, WeeFIM and PROMIS (Proxy/Pediatric) cover these areas more comprehensively than FMS and FAQ, which focus primarily on walking. However, clinicians should also be aware that transfer subscales (e.g., toilet and tub/shower) emphasize assistance levels rather than how tasks are accomplished or whether compensatory strategies are used. Similarly, PROMIS (Proxy/Pediatric) focused only on the difficulty of standing.

These findings also have implications for researchers seeking to refine or develop new mobility measures for AMC. Future work should consider revising existing tools by adding qualifiers, integrating missing concepts, or developing new measures that reflect the full spectrum of mobility challenges. This process should involve participatory methodologies, engaging children and youth with AMC and their families to capture their lived experiences and priorities (59).

### 4.1 Study limitations

First, the expert panel could be considered small, which may limit the generalizability of our findings. Future studies should consider including a larger number of participants, particularly individuals with lived experience, to further enhance the diversity of perspectives and the robustness of content validation. However, our panel represented diverse perspectives within the AMC community, including experienced researchers, individuals with AMC, and caregivers of children with AMC. Their clinical and lived experiences contributed critical insights, strengthening the relevance and applicability of the findings (59). Second, although our questionnaire followed established guidelines, its length may have contributed to expert fatigue, potentially resulting in faster, shorter, and more uniform responses to questions positioned later in the questionnaire (60). Future studies should consider refining the design to reduce redundancy and improve user experience, particularly for lay participants. Lastly, this study focused solely on content validity; other psychometric properties were not assessed. These measures have recently been evaluated for construct validity in a separate study using data from the North American AMC Registry, and the results are currently being prepared for publication.

## 5 Conclusions

The mobility measures examined in this study demonstrated good content validity overall. However, none of the tools fully addressed the breadth of mobility experiences in children with AMC. Incorporating missing concepts, such as environmental challenges, compensatory strategies, and pain, into existing or newly developed assessment tools is essential for providing a more holistic understanding of functional mobility. Doing so will help clinicians better evaluate intervention outcomes and ultimately improve the independence and quality of life for children living with AMC.

## Data Availability

The raw data supporting the conclusions of this article will be made available by the authors, without undue reservation.
