# Supplementary material for: Content validity of mobility measures in arthrogryposis multiplex congenita: engaging clinicians and people with lived experience
Source: Front Rehabil Sci. 2025 Aug 4;6:1576267. doi: 10.3389/fresc.2025.1576267 (PMC12358442; doi:10.3389/fresc.2025.1576267)
Supplement: Supplementary file 1 [file Datasheet1.pdf]

Supplementary Material

1 Supplementary Table 1

Themes (n=13) and meaningful concepts (n=50) based on content validity exercise

Themes	Meaningful concepts
**1. Walking**	1. Walking<br>2. Walking with assistance<br>3. Perform 75% or more of the effort to walk 150 feet<br>4. Perform less than 25% of the effort required for walking<br>5. Going for a walk of at least 15 mins
**2. Moving around**	6. Moving around in and between classes at school<br>7. Moving around long distances<br>8. Moving around short distances
**3. Wheelchair Mobility**	9. Perform 25% of the effort to travel with a wheelchair for 150 feet<br>10. Perform 75% of the effort to travel with a wheelchair for 150 feet<br>11. Travel with a wheelchair
**4. Crawling**	12. Crawling
**5. Stair navigation**	13. Going up and down stairs<br>14. Go up and down fewer than (4) stairs<br>15. Go up and down (4) stairs<br>16. Go up and down (12-14) stairs<br>17. Climbing<br>18. Perform 75% or more of the effort to go up and down 12–14 stairs<br>19. Walk upstairs without holding on to anything
**6. Basic Movements and Postures**	20. Getting up from the floor<br>21. Standing on tiptoes<br>22. Standing without help<br>23. Running<br>24. Sitting
**7. Physical Activities and Recreation**	25. Sports and exercise<br>26. Do the activities they enjoy<br>27. Keep up playing with other kids

**8. Transfer Tasks**	28. Transfer<br>29. Perform 25% or more of transfer tasks<br>30. Perform 50% or more of transfer tasks<br>31. Perform 75% or more of transfer tasks
**9. Assistance and Independence**	32. Need help<br>33. Assistance from 1 helper<br>34. Assistance from 2 helpers<br>35. Need of assistive device<br>36. No need for assistance
**10. Household Activities and Personal Errands**	37. Doing chores<br>38. Heavy work around the house<br>39. Moderate housework<br>40. Lifting or carrying groceries<br>41. Run errands and shopping
**11. Generic "Physical Labor & Health"**	42. Doing two hours of physical labor<br>43. Physical ability<br>44. Physical health
**12. Time and Effort**	45. Time to go up and down<br>46. Time to transfer<br>47. Normal pace<br>48. Distance
**13. Miscellaneous**	49. Moving legs<br>50. Safety

2 Supplementary Table 2

Content Validity index of the mobility measures

**Measure**		**Relevance to the construct**				**Relevance to the population**			
	Item	**Experts in Agreement**	**I-CVI**	**UA**	**k***	**Experts in Agreement**	**I-CVI**	**UA**	**k***
**FMS**	1	7	1	1	1	7	1	1	1
	2	7	1	1	1	7	1	1	1
	3	7	1	1	1	7	1	1	1
		S-CVI	1		1	S-CVI	1		

			S-CVI/UA	1			S-CVI/UA	1	
**FAQ**	1	7	1	1	1	7	1	1	1
		S-CVI	1			S-CVI	1		
			S-CVI/UA	1			S-CVI/UA	1	
**WeeFIM (Mobility)**									
Chair, Wheelchair	1	6	0.86	0	0.85	6	0.86	0	0.85
	2	6	0.86	0	0.85	6	0.86	0	0.85
	3	6	0.86	0	0.85	6	0.86	0	0.85
	4	6	0.86	0	0.85	6	0.86	0	0.85
	5	6	0.86	0	0.85	6	0.86	0	0.85
	6	6	0.86	0	0.85	6	0.86	0	0.85
		Sub-CVI	0.86*			Sub-CVI	0.86*		
			S-CVI/UA	0			S-CVI/UA	0	
Toilet	1	7	1	1	1	6	0.86	0	0.85
	2	6	0.86	0	0.85	7	1	1	1
	3	7	1	1	1	6	0.86	0	0.85
	4	6	0.86	0	0.85	6	0.86	0	0.85
	5	6	0.86	0	0.85	6	0.86	0	0.85
	6	6	0.86	0	0.85	6	0.86	0	0.85

		Sub-CVI	0.90			Sub-CVI	0.88*		
			S-CVI/UA	0.33			S-CVI/UA	0.17	
Tub, Shower	1	7	1	1	1	6	0.86	0	0.85
	2	6	0.86	0	0.85	7	1	1	1
	3	7	1	1	1	6	0.86	0	0.85
	4	6	0.86	0	0.85	6	0.86	0	0.85
	5	6	0.86	0	0.85	6	0.86	0	0.85
	6	6	0.86	0	0.85	6	0.86	0	0.85
		Sub-CVI	0.90			Sub-CVI	0.88*		
			S-CVI/UA	0.33			S-CVI/UA	0.17	
Walk	1	6	0.86	0	0.85	7	1	1	1
	2	7	1	1	1	6	0.86	0	0.85
	3	7	1	1	1	7	1	1	1
	4	7	1	1	1	6	0.86	0	0.85
	5	6	0.86	0	0.85	6	0.86	0	0.85
	6	7	1	1	1	6	0.86	0	0.85
	7	6	0.86	0	0.85	6	0.86	0	0.85
		Sub-CVI	0.94			Sub-CVI	0.90		
			S-CVI/UA	0.57			S-CVI/UA	0.29	

Wheelchair	1	7	1	1	1	6	0.86	0	0.85
	2	6	0.86	0	0.85	6	0.86	0	0.85
	3	6	0.86	0	0.85	6	0.86	0	0.85
	4	6	0.86	0	0.85	6	0.86	0	0.85
	5	6	0.86	0	0.85	6	0.86	0	0.85
	6	6	0.86	0	0.85	6	0.86	0	0.85
		Sub-CVI	0.88*			Sub-CVI	0.86*		
			S-CVI/UA	0.17			S-CVI/UA	0	
Crawl	1	7	1	1	1	6	0.86	0	0.85
	2	7	1	1	1	7	1	1	1
	3	7	1	1	1	7	1	1	1
		Sub-CVI	1			Sub-CVI	0.95		
			S-CVI/UA	1			S-CVI/UA	0.67	
Stairs	1	7	1	1	1	7	1	1	1
	2	7	1	1	1	7	1	1	1
	3	7	1	1	1	6	0.86	0	0.85
	4	7	1	1	1	6	0.86	0	0.85
	5	6	0.86	0	0.85	6	0.86	0	0.85
	6	6	0.86	0	0.85	6	0.86	0	0.85

	7	6	0.86	0	0.85	6	0.86	0	0.85
		Sub-CVI	0.94			Sub-CVI	0.90		
			S-CVI/UA	0.57			S-CVI/UA	0.29	
		S-CVI	0.91			S-CVI	0.89*		
**PROMIS (Mobility)**									
Parent Proxy (5-17) Mobility	1	6	0.86	0	0.85	6	0.86	0	0.85
	2	7	1	1	1	7	1	1	1
	3	6	0.86	0	0.85	7	1	1	1
	4	6	0.86	0	0.85	7	1	1	1
	5	7	1	1	1	7	1	1	1
	6	7	1	1	1	6	0.86	0	0.85
	7	7	1	1	1	6	0.86	0	0.85
	8	6	0.86	0	0.85	7	1	1	1
		S-CVI	0.93			S-CVI	0.95		
			S-CVI/UA	0.5			S-CVI/UA	0.625	
Pediatric (8-17) Mobility	1	6	0.86	0	0.85	6	0.86	0	0.85
	2	7	1	1	1	7	1	1	1
	3	6	0.86	0	0.85	7	1	1	1
	4	6	0.86	0	0.85	7	1	1	1

	5	7	1	1	1	7	1	1	1
	6	7	1	1	1	6	0.86	0	0.85
	7	7	1	1	1	6	0.86	0	0.85
	8	6	0.86	0	0.85	7	1	1	1
		S-CVI	0.93			S-CVI	0.95		
			S-CVI/UA	0.5			S-CVI/UA	0.625	
Young adult (18-21) Physical Function	1	6	0.86	0	0.85	6	0.86	0	0.85
	2	7	1	1	1	6	0.86	0	0.85
	3	7	1	1	1	7	1	1	1
	4	6	0.86	0	0.85	7	1	1	1
	5	5	0.71*	0	0.66	6	0.86	0	0.85
	6	5	0.71*	0	0.66	6	0.86	0	0.85
	7	5	0.71*	0	0.66	6	0.86	0	0.85
	8	5	0.71*	0	0.66	6	0.86	0	0.85
		S-CVI	0.82*			S-CVI	0.89*		
			S-CVI/UA	0.25			S-CVI/UA	0.25	

I-CVI: Item Content Validity index, S-CVI: Scale Content Validity index, Sub-CVI: Subscale CVI, UA: Universal Agreement, k*: The modified kappa, * values less than the recommended, FMS: Functional Mobility Scale, FAQ: Gillette Functional Assessment Questionnaire, WeeFIM: Functional Independence Measure for Children, PROMIS: Patient-Reported Outcomes Measurement Information System.
